# Individual and Area-level Factors Contributing to the Geographic Variation in Ambulatory Care Sensitive Conditions in Finland: A Register-based Study

**DOI:** 10.1097/MLR.0000000000001454

**Published:** 2020-11-16

**Authors:** Markku Satokangas, Martti Arffman, Harri Antikainen, Alastair H. Leyland, Ilmo Keskimäki

**Affiliations:** * Department of General Practice and Primary Health Care, Network of Academic Health Centres, University of Helsinki; † Service System Research Unit, Finnish Institute for Health and Welfare, Helsinki; ‡ Geography Research Unit, University of Oulu, Oulu, Finland; § MRC/CSO Social and Public Health Sciences Unit, University of Glasgow, Glasgow, Scotland; ∥ Faculty of Social Sciences, Tampere University, Tampere, Finland

**Keywords:** health services research, multilevel modelling, preventable hospitalizations, primary care

## Abstract

**Background:**

Measuring primary health care (PHC) performance through hospitalizations for ambulatory care sensitive conditions (ACSCs) remains controversial—recent cross-sectional research claims that its geographic variation associates more with individual socioeconomic position (SEP) and health status than PHC supply.

**Objectives:**

To clarify the usage of ACSCs as a PHC performance indicator by quantifying how disease burden, both PHC and hospital supply and spatial access contribute over time to geographic variation in Finland when individual SEP and comorbidities were adjusted for.

**Methods:**

The Finnish Care Register for Health Care provided hospitalizations for ACSCs (divided further into subgroups of acute, chronic, and vaccine-preventable causes) in 2011–2017. With 3-level nested multilevel Poisson models—individuals, PHC authorities, and hospital authorities—we estimated the proportion of the variance in ACSCs explained by selected factors at 3 time periods.

**Results:**

In age-adjusted and sex-adjusted analysis of total ACSCs the variances between hospital authorities was nearly twice that between PHC authorities. Individual SEP and comorbidities explained 19%–30% of the variance between PHC authorities and 25%–36% between hospital authorities; and area-level disease burden and arrangement and usage of hospital care a further 14%–16% and 32%–33%—evening out the unexplained variances between PHC and hospital authorities.

**Conclusions:**

Alongside individual factors, areas’ disease burden and factors related to hospital care explained the excess variances in ACSCs captured by hospital authorities. Our consistent findings over time suggest that the local strain on health care and the regional arrangement of hospital services affect ACSCs—necessitating caution when comparing areas’ PHC performance through ACSCs.

## Abstract 2

Supplemental Digital Content is available in the text.

Hospitalizations for ambulatory care sensitive conditions (ACSC) is among the most commonly used proxy indicators to measure primary health care (PHC) performance.^1^ ACSCs have been suggested as potentially avoidable by well-functioning PHC.^2^ As equity in delivery of health care can be assessed by measuring geographic variation in different medical practices,^3^ delivery of PHC can be assessed through geographic variation in ACSCs. Variation in ACSC is a common phenomenon,^4^ but it is driven more by individual socioeconomic position (SEP) and health status than general practitioner (GP) workload.^5^ Thus, the link between ACSCs and PHC performance remains controversial (Table 1).

**TABLE 1:** Glossary

ACSC	Ambulatory care sensitive conditions
COPD	Chronic obstructive pulmonary disease
GP	General practitioner
ICD-10	International Classification of Diseases 10th revision
IRR	Incidence rate ratio
MRR	Median rate ratio
PHC	Primary health care
PCV	Proportional change in variance
SEP	Socioeconomic position

ACSCs seem to associate with increased health needs of individuals.^6^,^7^ From the patients’ perspective ACSCs result from nonadherence to treatment; for example, due to combined lack of support and mental health issues.^8^ This helps to understand why ACSCs are reduced by better patient support through specified family physicians,^9^ payment models that reward comprehensive care^10^ and continuity of care.^11^,^12^ However, ACSC rates do not mirror the results of PHC clinical quality indicators^13^—and their connection with the number of GPs remains inconsistent.^9^,^14^

Further, some individual and population characteristics contributing to variation in ACSCs might be partially addressable by PHC policies. Although the individual socioeconomic gradient in ACSC rates disfavors the poorest, these rates still differ depending on the incomes of residential areas.^15^ And while individual comorbidities are a major predictor of ACSCs,^16^,^17^ even area-level disease prevalence explains variation in ACSC rates.^18^ Shorter travel time to PHC reduces ACSCs rates,^9^,^19^ while shorter travel time to hospitals,^19^ high rurality,^20^ and high hospital bed supply^12^,^21^,^22^ increase them.

Few studies have assessed over time the development of geographic distribution^23^,^24^ or variation in ACSC rates,^25^,^26^ but describe these only with area-level factors.^23^,^24^ It is still unclear how a comprehensive array of area-level factors over time contributes to geographic variation in ACSCs, when individual SEP and health status are adjusted for.

## FINNISH CONTEXT

The mainly tax-funded Finnish health care system offers a good framework to assess variation in ACSCs, comprising universal access, hierarchical structure and a long tradition of collecting individual hospitalization data. Finland has a strong public PHC where GPs operate as gatekeepers to specialist care provided by public hospitals.^27^ PHC is provided through ∼150 health centers,^28^ which act also as community hospitals by providing inpatient care in GP led wards. Hospital care is provided through 20 hospital districts—each having mainly a single 24/7 emergency hospital. An individual receives PHC services from a single health center, which receives its specialist care through a single hospital district. However, occupational and private health care offer alternative routes to GP and specialist care outpatient consultations.

## METHODS

### Outcome Variable

The Finnish Care Register for Health Care provided individual hospitalizations for the total Finnish population aged 20 years or more in 2011–2017. Of these we identified ACSCs using the UK definition^29^—with an addition of unspecified pneumonia (ICD-10 diagnosis code J18.9) as used previously in Finland.^23^ Further, we divided ACSCs into subgroups of acute, chronic, and vaccine-preventable (Supplemental Digital Content 1, http://links.lww.com/MLR/C130). Competent authorities linked hospitalization data into individual sociodemographic data. We formed annual cohorts and applied municipality of residence to allocate individuals into health center areas according to arrangement of PHC; and into hospital districts according to arrangement of hospital care. To account for hospital transfers we combined hospitalizations that occurred within 1 day of each other.

With these cohorts we asked: (1) how geographic variance in ACSCs was distributed between PHC and hospital care; (2) which factors predicted ACSCs; (3) which area-level factors explained variance in ACSCs when analyzed separately; (4) what were the relative proportions of variance explained by area-level factors after adjustment for individual factors; and (5) how the heterogeneity in risk of ACSCs developed with each added factor?

### Individual Factors

The annual FOLK databases provided data for sex, age, municipality of residence, and household income for each individual with ACSCs. For these individuals we selected 5 comorbidities [chronic heart failure, chronic obstructive pulmonary disease (COPD), diabetes, hypertension, and dementia] as suggested by Saver et al^16^ from inpatient hospitalizations and specialist outpatient visits during the previous 5 years.

### Area-level Factors

We chose area-level factors previously suggested to affect ACSCs, such as disease prevalence^18^ and hospital supply.^12^ These factors were allocated to health center areas and further categorized for the purposes of reporting—as summarized in Table 2. We categorized the proportion of the population aged 65 and above receiving pensioner’s care allowance as disease burden because it captures both disease prevalence and functional limitations. It is granted when a chronic disease or disability limits daily living activities.

**TABLE 2:** The Categories of Individual and Area-level Factors Added Into the Models Estimating the Geographic Variance of ACSCs

Added to	Category	Factors	Hypothesized Pathway for Risk of ACSC Hospitalization	Data Obtained From
Model 1	Individual demographics	Age and sex	Null model	FOLK database maintained by Statistics Finland
Model 2	Individual SEP and health status	Household incomes	Lower SEP predisposes to material deprivation and possibly to poorer care,^30^ for example through communication mismatch with GPs^31^	FOLK database maintained by Statistics Finland
		Number of comorbidities	Multimorbidity complicates treatments and associates with acute diseases leading to hospitalizations^32^	The Care Register for Health Care maintained by Finnish Institute of Health and Welfare
Model 3	Area-level disease burden	Proportion of population aged ≥65 receiving pensioner’s care allowance (%)	Higher proportion of multimorbid elderly (with limitations of activities in daily living) within an area strain health care, possibly lowering the threshold of hospital utilization	Statistical database Kelasto maintained by Social Insurance Institution of Finland^33^
Model 4	Area-level arrangement and usage of hospital care	Proportion of ACSCs occurring in GP led wards of all ACSCs (%)	GP led wards are likely to have less constrictive intake criteria than specialist care	Aggregated from the Care Register for Health Care maintained by Finnish Institute of Health and Welfare
		Rate of hospital bed utilization in specialist health care	Available hospital beds promote utilization of hospital care^34^	
Model 5	Area-level distance to health services	Populations’ average distance to health center (km)	Long distance to health services might cause delay and lead to disease exacerbation	Road and street network data provided by both Esri Finland and the Finnish Transport Agency (Digiroad database)
		Populations’ average distance to emergency hospital (km)		
Model 6	Other area-level factors related to health services	Number of GPs per 1000 inhabitants	Fewer GPs represent poorer availability of care	General Practitioner survey provided by Finnish Medical Association
		Income median (€)	Area’s wealth might affect the care of multimorbid patients,^35^ differences in provision of primary care^15^	Aggregated from the FOLK database maintained by Statistics Finland

The subsequent models include all factors from the previous ones. All area-level factors were allocated to health center areas.

ACSC indicates ambulatory care sensitive conditions; GP, general practitioner; SEP, socioeconomic position.

### Statistical Methods

Age-standardized rates were calculated using the direct standardization.^36^ We built on the analysis strategy presented by Falster et al^5^ and allocated the annual cohorts to 3-level Poisson multilevel models: individuals were nested within 131 health center areas, which were in turn nested within 20 hospital districts. We analyzed separate models for total ACSCs and each of the 3 ACSC subgroups in 3 consecutive time periods: 2011–2012, 2013–2014, and 2015–2017. The total Finnish population of comparable age was applied as the population at risk and added into the models as an offset (range, 4020–519,853 in health centers and 22,410–1,288,747 in hospital districts).

By first analyzing an age-adjusted and sex-adjusted model (model 1) we estimated the random parameters (σ^2^) representing the variance at health center and hospital district levels to which we compared all subsequent models. We added the factors stepwise into the models; and measured the effects of each addition with the proportional change in variance (PCV).^37^ To analyze which factors predicted ACSCs we calculated incidence rate ratios (IRR). Finally, we calculated the areas’ heterogeneity in risk of ACSCs with median rate ratios.^38^ The statistical analyses were performed with R, release versions 3.5.1^39^—using the Laplace approximation method.^40^

## RESULTS

We observed 729,008 ACSCs in Finland in 2011–2017 (Table 3). The age-standardized ACSC rate in the adult population decreased from 2.67 (95% confidence interval, 2.66–2.68) per 100 person-years in 2011–2012 to 2.57 (2.56–2.58) in 2015–2017. In the age-adjusted and sex-adjusted models—model 1—of total ACSCs the hospital district level variance was approximately twice that of the health center level. These variances decreased slightly over time at both area levels, although the decrease was more pronounced at hospital district level.

**TABLE 3:** Cohort Characteristics for All Hospitalizations for ACSCs and Average ACSC Rates per 100 Person-Years in 3 Studied Time Periods

	2011–2012			2013–2014			2015–2017		
Variables	Range of Area-level Factors	Persons	ACSC Rate	Range of Area-level Factors	Persons	ACSC Rate	Range of Area-level Factors	Persons	ACSC Rate
Total study population		4,203,024	2.5		4,254,995	2.5		4,388,766	2.6
Age (y)									
20–54		1,449,327	0.6		1,474,922	0.6		1,573,093	0.6
55–64		1,463,115	1.1		1,428,219	1.0		1,403,760	1.0
65–74		672,900	2.6		708,863	2.5		739,912	2.5
75–84		392,232	6.1		406,370	5.8		426,180	5.8
85+		225,450	15.9		236,621	15.6		245,821	16.2
Sex									
Males		2,043,104	2.6		2,071,126	2.5		2,143,146	2.7
Females		2,159,920	2.4		2,183,869	2.4		2,245,620	2.5
Income									
Quintile 1		855,827	4.7		865,091	4.5		904,007	4.4
Quintile 2		812,472	3.9		824,835	3.8		845,825	4.1
Quintile 3		803,963	2.1		815,350	2.0		842,794	2.3
Quintile 4		836,117	1.3		845,743	1.3		870,886	1.4
Quintile 5		894,479	0.9		903,808	0.9		925,084	0.9
No. comorbidities									
0		3,829,145	1.3		3,853,135	1.2		3,961,902	1.3
1		285,115	10.5		301,763	9.8		316,921	9.8
2		74,391	23.8		84,120	22.5		92,130	22.4
3+		14,373	54.7		15,977	52.6		17,813	55.4
Proportion of population aged ≥65 receiving pensioner’s care allowance (%)									
Tercile 1	8.3–16.7	1,763,672	1.9	9.0–15.8	1,827,338	1.9	8.1–14.6	1,877,242	2.0
Tercile 2	16.8–19.5	1,562,636	2.7	15.9–18.5	1,550,841	2.6	14.7–17.5	1,664,597	2.7
Tercile 3	19.6–25.6	876,716	3.5	18.6–25.0	876,816	3.3	17.6–24.0	846,927	3.5
Proportion of ACSCs occurring in GP led wards of all ACSCs (%)									
Tercile 1	32.9–25.4	1,842,453	2.3	1.1–29.7	1,953,356	2.3	0.5–26.8	2,454,779	2.2
Tercile 2	25.5–41.2	1,162,415	2.6	29.8–42.5	1,581,758	2.2	26.9–45.3	1,016,471	2.9
Tercile 3	41.3–72.9	1,198,156	2.8	42.6–73.7	719,881	3.4	45.4–73.2	917,516	3.3
Rate of hospital bed utilization in specialist health care									
Tercile 1	0.13–0.23	2,395,000	2.0	0.14–0.22	2,362,852	1.9	0.14–0.21	2,400,369	2.0
Tercile 2	0.24–0.28	1,204,534	3.0	0.23–0.27	1,329,090	2.8	0.22–0.26	1,285,578	2.9
Tercile 3	0.29–0.39	603,490	3.9	0.28–0.41	563,053	3.9	0.27–0.48	702,819	3.9
Populations’ average distance to health center (km)									
Tercile 1	1.2–4.7	2,374,959	2.1	1.2–4.6	2,410,214	2.0	1.2–4.8	2,517,459	2.1
Tercile 2	4.8–6.6	1,070,273	3.0	4.7–6.6	1,039,132	2.9	4.8–6.6	1,026,532	3.0
Tercile 3	6.7–26.4	757,792	3.4	6.7–26.8	805,649	3.2	6.7–26.9	844,775	3.3
Populations’ average distance to emergency hospital (km)									
Tercile 1	2.8–20.8	2,673,218	2.2	2.8–21.1	2,741,170	2.1	2.8–21.8	2,900,462	2.3
Tercile 2	20.9–45.8	957,014	2.7	21.2–45.8	948,449	2.6	21.9–51.3	941,334	2.6
Tercile 3	45.9–331.1	572,792	3.9	45.9–331.1	565,376	3.8	51.4–331.1	546,970	3.9
No. GPs per 1000 inhabitants									
Tercile 1 (median)*	0.58	2,281,069	2.3	0.61	2,500,773	2.2	0.59	2,803,283	2.3
Tercile 2 (median)*	0.71	1,265,989	2.7	0.74	932,620	2.8	0.74	877,856	2.8
Tercile 3 (median)*	0.82	655,966	3.2	0.84	821,602	3.0	0.85	707,627	3.4
Income median (€)									
Tercile 1	17,900–20,600	772,433	3.7	19,000–21,900	722,894	3.6	20,000–22,800	868,618	3.4
Tercile 2	20,700–22,200	1,695,028	2.6	22,000–23,700	1,767,878	2.6	22,800–24,400	1,695,119	2.8
Tercile 3	22,300–36,800	1,735,563	1.9	23,800–38,600	1,764,223	1.8	24,500–40,600	1,825,029	2.0

* We had no permission to report the range for number of GPs acquired from the General Practitioner survey.

ACSC indicates ambulatory care sensitive conditions; GP, general practitioner.

In the final models (model 6) male sex, higher age, lower income, and higher number of comorbidities associated with higher IRRs in total ACSCs and all ACSC subgroups (Table 4). Area-level higher disease burden, hospital bed utilization rate, and median income were associated with higher IRRs in total ACSCs in all time periods—but a higher proportion of ACSCs in GP led wards only in 2013–2014 (Supplemental Digital Content 1, http://links.lww.com/MLR/C130).

**TABLE 4:** IRRs of Individual and Area-level Factors Significantly Associated With Total ACSCs and ACSC Subgroups in Finland in 2015–2017; From the Multilevel Poisson Models Adjusted Simultaneously for All Individual and Area-level Factors (Model 6)

	Total		Acute		Chronic		Vaccine-preventable	
Variables	IRR (95% CI)	*P*	IRR (95% CI)	*P*	IRR (95% CI)	*P*	IRR (95% CI)	*P*
Sex								
Male	1.00		1.00		1.00		1.00	
Female	0.74 (0.73–0.74)	**<0.001**	0.95 (0.93–0.96)	**<0.001**	0.75 (0.74–0.76)	**<0.001**	0.60 (0.59–0.61)	**<0.001**
Age (y)								
20–54	1.00		1.00		1.00		1.00	
55–64	1.77 (1.74–1.80)	**<0.001**	1.11 (1.08–1.14)	**<0.001**	3.73 (3.59–3.88)	**<0.001**	2.16 (2.09–2.22)	**<0.001**
65–74	3.58 (3.52–3.63)	**<0.001**	1.66 (1.62–1.70)	**<0.001**	8.95 (8.62–9.29)	**<0.001**	4.88 (4.74–5.02)	**<0.001**
75–84	6.28 (6.18–6.37)	**<0.001**	2.47 (2.41–2.53)	**<0.001**	16.12 (15.54–16.73)	**<0.001**	9.20 (8.95–9.46)	**<0.001**
85+	12.06 (11.88–12.25)	**<0.001**	4.71 (4.60–4.83)	**<0.001**	28.69 (27.66–29.77)	**<0.001**	19.66 (19.13–20.21)	**<0.001**
Income quintile								
Lowest	1.00		1.00		1.00		1.00	
2	0.78 (0.77–0.78)	**<0.001**	0.74 (0.72–0.75)	**<0.001**	0.77 (0.76–0.78)	**<0.001**	0.80 (0.79–0.82)	**<0.001**
3	0.65 (0.64–0.65)	**<0.001**	0.63 (0.61–0.64)	**<0.001**	0.62 (0.61–0.63)	**<0.001**	0.69 (0.67–0.70)	**<0.001**
4	0.54 (0.53–0.55)	**<0.001**	0.53 (0.51–0.54)	**<0.001**	0.50 (0.49–0.51)	**<0.001**	0.59 (0.58–0.60)	**<0.001**
Highest	0.43 (0.43–0.44)	**<0.001**	0.44 (0.43–0.45)	**<0.001**	0.38 (0.37–0.39)	**<0.001**	0.48 (0.47–0.49)	**<0.001**
No. comorbidities: 0–5 (+1 comorbidity)	2.33 (2.32–2.33)	**<0.001**	1.89 (1.88–1.91)	**<0.001**	2.80 (2.79–2.81)	**<0.001**	2.01 (2.00–2.02)	**<0.001**
Proportion of population aged 65+ receiving pensioner’s care allowance (+1 SD)	1.06 (1.02–1.11)	**0.008**	1.12 (1.05–1.19)	**<0.001**	1.00 (0.95–1.06)	0.867	1.09 (1.04–1.15)	**0.001**
Proportion of ACSCs occurring in GP led wards of all ACSC hospitalizations (+1 SD)	1.03 (1.00–1.07)	**0.024**	1.01 (0.97–1.05)	0.622	1.04 (1.00–1.07)	0.060	1.06 (1.02–1.10)	**0.002**
Rate of hospital bed utilization in specialist health care (+1 SD)	1.08 (1.04–1.12)	**<0.001**	1.08 (1.03–1.14)	**0.002**	1.12 (1.07–1.17)	**<0.001**	1.02 (0.98–1.07)	0.325
Income median (+1 SD)	1.07 (1.03–1.11)	**0.001**	1.10 (1.05–1.16)	**<0.001**	1.05 (1.01–1.10)	**0.029**	1.06 (1.01–1.11)	**0.020**

The IRR of area-level distances to health services and number of GPs did not significantly associate with ACSCs. Over time all these associations were stable as shown in Supplementary Digital Content 1 (http://links.lww.com/MLR/C130). A bold font indicate statistically significant *P* values.

ACSC indicates ambulatory care sensitive conditions; CI, confidence interval; GP, general practitioner; IRR, incidence rate ratios.

Each area-level factor explained variation in ACSCs when analyzed separately (Supplemental Digital Content 1, http://links.lww.com/MLR/C130). For total ACSCs our final model explained a little less than half (PCV=39.8%–52.1%) the health center level variance and almost two thirds (PCV=61.1%–74.2%) the hospital district level variance. The highest PCV occurred in 2011–2012 from when it decreased over time along with the variances in age-adjusted and sex-adjusted models. Although individual SEP and health status explained 18.7%–29.9% of health center level variance and 24.6%–35.7% of hospital district level variance, a combination of area-level disease burden and both arrangement and usage of hospital care accounted for an additional 14.1%–16.2% and 32.4%–33.0% of the respective variances. And while the distance to health services and number of GPs did not add much to the models, the areas’ median income accounted for an additional 2.7%–6.5% and 4.2%–7.6% of these variances. Areas had some heterogeneity in risk of ACSCs which decreased with subsequent models (Table 5).

**TABLE 5:** Variance in Age-adjusted and Sex-adjusted Model (Model 1) in Total ACSCs and ACSC Subgroups Between HC Areas and HD in Finland at 3 Consecutive Time Periods

	2011–2012						2013–2014						2015–2017					
	HC			HD			HC			HD			HC			HD		
Models	σ^2^	PCV (%)	MRR	σ^2^	PCV (%)	MRR	σ^2^	PCV (%)	MRR	σ^2^	PCV (%)	MRR	σ^2^	PCV (%)	MRR	σ^2^	PCV (%)	MRR
Total ACSCs																		
Model 1	0.023	—	1.16	0.050	—	1.24	0.020	—	1.14	0.034	—	1.19	0.019	—	1.14	0.037	—	1.20
Model 2	0.016	29.9	1.13	0.032	35.7	1.19	0.014	27.9	1.12	0.026	24.6	1.16	0.016	18.7	1.13	0.025	31.0	1.16
Model 3	0.014	37.3	1.12	0.018	63.3	1.14	0.013	35.2	1.11	0.018	46.5	1.14	0.015	21.7	1.12	0.019	48.3	1.14
Model 4	0.013	44.0	1.11	0.016	68.1	1.13	0.011	44.1	1.10	0.014	57.3	1.12	0.013	34.3	1.11	0.013	64.0	1.12
Model 5	0.013	45.6	1.11	0.017	66.6	1.13	0.011	44.7	1.10	0.015	54.8	1.13	0.013	34.5	1.11	0.013	63.3	1.12
Model 6	0.011	52.1	1.11	0.013	74.2	1.11	0.010	47.5	1.10	0.013	61.1	1.12	0.012	39.8	1.11	0.012	67.4	1.11
Acute ACSCs																		
Model 1	0.027	—	1.17	0.038	—	1.21	0.025	—	1.16	0.035	—	1.20	0.026	—	1.17	0.055	—	1.25
Model 2	0.027	0.2	1.17	0.029	24.2	1.18	0.022	9.6	1.15	0.026	25.9	1.17	0.024	8.1	1.16	0.041	26.2	1.21
Model 3	0.027	0.8	1.17	0.018	52.9	1.14	0.022	11.8	1.15	0.016	53.6	1.13	0.023	10.8	1.16	0.029	46.8	1.18
Model 4	0.026	3.8	1.17	0.017	56.8	1.13	0.021	16.0	1.15	0.013	62.4	1.12	0.022	12.7	1.15	0.023	59.0	1.15
Model 5	0.025	6.7	1.16	0.017	57.0	1.13	0.020	17.8	1.15	0.012	65.6	1.12	0.022	13.4	1.15	0.022	59.5	1.15
Model 6	0.021	21.5	1.15	0.013	66.1	1.11	0.019	21.8	1.14	0.010	71.4	1.10	0.020	22.7	1.14	0.019	65.7	1.14
Chronic ACSCs																		
Model 1	0.034	—	1.19	0.072	—	1.29	0.029	—	1.18	0.055	—	1.25	0.027	—	1.17	0.035	—	1.20
Model 2	0.024	28.9	1.16	0.049	32.6	1.23	0.025	13.4	1.16	0.044	21.5	1.22	0.023	13.9	1.16	0.026	27.6	1.16
Model 3	0.023	31.8	1.16	0.033	55.0	1.19	0.025	14.9	1.16	0.035	36.7	1.20	0.023	13.3	1.16	0.022	38.5	1.15
Model 4	0.020	41.9	1.14	0.027	63.1	1.17	0.021	29.7	1.15	0.026	53.3	1.17	0.018	34.7	1.13	0.016	55.3	1.13
Model 5	0.019	44.3	1.14	0.030	57.9	1.18	0.020	32.0	1.14	0.030	46.7	1.18	0.017	36.2	1.13	0.017	51.4	1.13
Model 6	0.018	45.6	1.14	0.028	61.4	1.17	0.020	32.3	1.14	0.027	51.2	1.17	0.017	38.5	1.13	0.016	53.5	1.13
Vaccine-preventable ACSCs																		
Model 1	0.024	—	1.16	0.033	—	1.19	0.026	—	1.17	0.021	—	1.15	0.024	—	1.16	0.037	—	1.20
Model 2	0.018	25.9	1.14	0.026	20.1	1.17	0.020	26.1	1.14	0.021	1.4	1.15	0.021	13.1	1.15	0.032	14.8	1.18
Model 3	0.014	43.0	1.12	0.019	42.2	1.14	0.016	40.1	1.13	0.019	9.6	1.14	0.020	19.9	1.14	0.026	31.0	1.16
Model 4	0.013	46.4	1.11	0.017	47.4	1.13	0.015	44.0	1.12	0.018	15.0	1.14	0.018	28.4	1.13	0.020	45.9	1.14
Model 5	0.013	46.6	1.11	0.017	48.7	1.13	0.015	44.1	1.12	0.018	15.2	1.14	0.018	28.4	1.13	0.020	46.0	1.14
Model 6	0.011	52.9	1.11	0.015	56.1	1.12	0.014	48.9	1.12	0.016	21.1	1.13	0.017	31.8	1.13	0.019	48.8	1.14

Each subsequent model builds on the previous one and adds a category of explanatory factors: individual socioeconomic position and health status (model 2), area-level disease burden (model 3), area-level arrangement and usage of hospital care (model 4), area-level distance to health services (model 5), and other area-level factors related to health services (model 6).

ACSCs indicates ambulatory care sensitive conditions; HC, health center; HD, hospital districts; MRR, median rate ratio; PCV, proportional change in variance, calculated as percentual decrease in variance between each model and model 1.

## DISCUSSION

This study analyzed how individual and area-level factors over time contributed to geographic variance in ACSCs between 2 nested levels of health service providers in Finland. Among total ACSCs our final models explained less than half (PCV=39.8%–52.1%) the variance between health center areas and almost two thirds (PCV=61.1%–74.2%) between hospital districts. Even after adjusting for individual SEP and health status, area-level disease burden and both arrangement and usage of hospital care still explained 14.1%–16.2% and 32.4%–33.0% of these variances. The proportions explained were consistent over time. In age-adjusted and sex-adjusted models hospital districts showed more variation than health center areas—a disparity which evened out after adjusting for the studied factors. This suggest that variation in age-standardized and sex-standardized ACSC rates could be driven more by factors related to hospital services rather than PHC.

Our findings support the previous studies stating that variation in ACSCs reflects health status,^5^,^16^–^18^,^41^ SEP,^5^,^12^,^15^,^21^ and factors related to hospital care.^12^,^19^,^21^,^42^–^44^ However, this study emphasized that these factors explained more of the variance occurring in hospital district level than in health center level.

The finding for area-level disease burden—a combination of disease prevalence and functional limitations—adds to earlier knowledge.^18^ It explained additional variance independent of individual SEP and comorbidities. We suggest caution when interpreting ACSC rates adjusted with only disease prevalence—especially if this factor is used as a proxy for individual health status. We had a few possible hypotheses for this independent effect. At the hospital district level, it might reflect either different admission criteria between hospitals or systematically insufficient capacity of PHC to answer the high morbidity and disabilities. For health centers, it might reflect inadequate response of PHC in some areas; a possible link between ACSCs and PHC performance.

The finding that local GP led wards maintained variation in ACSCs was consistent with previous studies.^42^,^43^ Countries applying ACSC rates as PHC performance indicators should consider if their arrangement of hospital care affects these rates. ACSCs also reflected areas’ overall tendency for hospital utilization, suggesting unnecessary use of available hospital supply^34^ rather than different GP referral practices.^45^ The effects of distances to health services and the number of GPs were captured by other factors—such as the arrangement of hospital care. Our finding that higher area-level income predicted higher ACSCs contradicts the previously reported low ACSC rates in wealthy areas.^15^ These lower rates might not reflect better performing PHC, but rather other factors such as population’s favorable health status.

### Strengths and Limitations

The main strength of this study was that we were able to apply nested multilevel Poisson model to distinguish the variance in ACSCs between PHC and hospital care. Further, the individual hospitalization data used are of good quality^46^—and their comprehensive usage ensured the generalizability of our results in Finnish context. The relative proportions will differ when analyzed elsewhere, but the phenomena behind the analyzed factors are transferrable internationally.

Our study was limited by the lack of individual-level survey data to enrich the applied registers. Moreover, we did not analyze the effect of alternative routes to physician consultations in Finland: occupational and private health care—but believe that including individual and area-level incomes account for them. As the geographic diagnosis coverage in Finnish Register of PHC visits was partial,^47^ we had to collect individual comorbidities from both specialist care outpatient visits and hospital discharges.

## CONCLUSIONS

This study observed that administrative areas’ disease burden and hospital care utilization patterns contributed to geographic variation in ACSCs—potential links to PHC performance. This followed adjusting ACSCs for individual SEP and health status, as well as for the country-specific arrangement of hospital care. Countries measuring PHC performance with ACSC rates should (1) consider that these rates might be driven more by hospital care than PHC; (2) interpret prevalence adjusted rates with caution; and (3) consider that the arrangement of their hospital care might affect not only ACSC rates, but also other factors thought to reflect only the provision of PHC services.

## Supplementary Material

SUPPLEMENTARY MATERIAL

Supplemental Digital Content is available for this article. Direct URL citations appear in the printed text and are provided in the HTML and PDF versions of this article on the journal's website, www.lww-medicalcare.com.

## References

[1] Billings J Anderson GM Newman LS Recent findings on preventable hospitalizations. Health Aff. 1996;15:239–249. 10.1377/hlthaff.15.3.239 8854530

[2] Billings J Zeitel L Lukomnik J Impact of socioeconomic status on hospital use in New York City. Health Aff. 1993;12:162–173. 10.1377/hlthaff.12.1.162 8509018

[3] Westert GP Groenewegen PP Boshuizen HC Medical practice variations in hospital care; time trends of a spatial phenomenon. Health Place. 2004;10:215–220. 15177196 10.1016/j.healthplace.2003.07.002

[4] Busby J Purdy S Hollingworth W A systematic review of the magnitude and cause of geographic variation in unplanned hospital admission rates and length of stay for ambulatory care sensitive conditions. BMC Health Serv Res. 2015;15:324. 26268576 10.1186/s12913-015-0964-3 PMC4535775

[5] Falster MO Jorm LR Douglas KA Sociodemographic and health characteristics, rather than primary care supply, are major drivers of geographic variation in preventable hospitalizations in Australia. Med Care. 2015;53:436–445. 25793270 10.1097/MLR.0000000000000342 PMC4396734

[6] Falster MO Jorm LR Leyland AH Visualising linked health data to explore health events around preventable hospitalisations in NSW Australia. BMJ Open. 2016;6:e012031. 10.1136/bmjopen-2016-012031 PMC5020859 27604087

[7] Vuik SI Fontana G Mayer E Do hospitalisations for ambulatory care sensitive conditions reflect low access to primary care? An observational cohort study of primary care usage prior to hospitalisation. BMJ Open. 2017;7:e015704. 10.1136/bmjopen-2016-015704 PMC5724125 28827243

[8] Longman JM Rix E Johnston JJ Ambulatory care sensitive chronic conditions: what can we learn from patients about the role of primary health care in preventing admissions? Aust J Prim Health. 2018;24:304–310. 10.1071/PY17191 30078392

[9] Carneiro CS Hospitalisation of ambulatory care sensitive conditions and access to primary care in Portugal. Public Health. 2018;165:117–124. 30390424 10.1016/j.puhe.2018.09.019

[10] Laberge M Wodchis WP Barnsley J Hospitalizations for ambulatory care sensitive conditions across primary care models in Ontario, Canada. Social Sci Med. 2017;181:24–33. 10.1016/j.socscimed.2017.03.040 28365547

[11] Barker I Steventon A Deeny SR Association between continuity of care in general practice and hospital admissions for ambulatory care sensitive conditions: cross sectional study of routinely collected, person level data. BMJ. 2017;356:j84. 28148478 10.1136/bmj.j84

[12] Busby J Purdy S Hollingworth W How do population, general practice and hospital factors influence ambulatory care sensitive admissions: a cross sectional study. BMC Fam Pract. 2017;18:67. 28545412 10.1186/s12875-017-0638-9 PMC5445441

[13] van der Pol M Olajide D Dusheiko M The impact of quality and accessibility of primary care on emergency admissions for a range of chronic ambulatory care sensitive conditions (ACSCs) in Scotland: longitudinal analysis. BMC Fam Pract. 2019;20:32. 30795737 10.1186/s12875-019-0921-z PMC6385424

[14] Chang C-H O’Malley AJ Goodman DC Association between temporal changes in primary care workforce and patient outcomes. Health Serv Res. 2017;52:634–655. 27256769 10.1111/1475-6773.12513 PMC5346500

[15] Lofqvist T Burstrom B Walander A Inequalities in avoidable hospitalisation by area income and the role of individual characteristics: a population-based register study in Stockholm County, Sweden. BMJ Qual Saf. 2014;23:206–214. 10.1136/bmjqs-2012-001715 24082149

[16] Saver BG Wang CY Dobie SA The central role of comorbidity in predicting ambulatory care sensitive hospitalizations. Eur J Public Health. 2014;24:66–72. 23543676 10.1093/eurpub/ckt019 PMC3901312

[17] Eggli Y Desquins B Seker E Comparing potentially avoidable hospitalization rates related to ambulatory care sensitive conditions in Switzerland: the need to refine the definition of health conditions and to adjust for population health status. BMC Health Serv Res. 2014;14:25. 24438689 10.1186/1472-6963-14-25 PMC3902189

[18] Pollmanns J Romano PS Weyermann M Impact of disease prevalence adjustment on hospitalization rates for chronic ambulatory care-sensitive conditions in Germany. Health Serv Res. 2018;53:1180–1202. 28332190 10.1111/1475-6773.12680 PMC5867184

[19] Huang Y Meyer P Jin L Spatial access to health care and elderly ambulatory care sensitive hospitalizations. Public Health. 2019;169:76–83. 30825835 10.1016/j.puhe.2019.01.005

[20] Burgdorf F Sundmacher L Potentially avoidable hospital admissions in Germany: an analysis of factors influencing rates of ambulatory care sensitive hospitalizations. Dtsch Arztebl Int. 2014;111:215–223. 24739884 10.3238/arztebl.2014.0215 PMC3991158

[21] Weeks WB Ventelou B Paraponaris A Rates of admission for ambulatory care sensitive conditions in France in 2009–2010: trends, geographic variation, costs, and an international comparison. Eur J Health Econ. 2016;17:453–470. 25951924 10.1007/s10198-015-0692-y

[22] Van Loenen T Faber MJ Westert GP The impact of primary care organization on avoidable hospital admissions for diabetes in 23 countries. Scand J Primary Health Care. 2016;34:5–12. 10.3109/02813432.2015.1132883 PMC4911022 26849246

[23] Satokangas M Lumme S Arffman M Trajectory modelling of ambulatory care sensitive conditions in Finland in 1996–2013: assessing the development of equity in primary health care through clustering of geographic areas—an observational retrospective study. BMC Health Serv Res. 2019;19:629. 31484530 10.1186/s12913-019-4449-7 PMC6727548

[24] Sheringham J Asaria M Barratt H Are some areas more equal than others? Socioeconomic inequality in potentially avoidable emergency hospital admissions within English local authority areas. J Health Serv Res Policy. 2017;22:83–90. 28429977 10.1177/1355819616679198 PMC5347357

[25] Manderbacka K Arffman M Satokangas M Regional variation of avoidable hospitalisations in a universal health care system: a register-based cohort study from Finland 1996−2013. BMJ Open. 2019;9:e029592. 10.1136/bmjopen-2019-029592 PMC6661699 31324684

[26] Librero J Ibañez-Beroiz B Peiró S Trends and area variations in potentially preventable admissions for COPD in Spain (2002–2013): a significant decline and convergence between areas. BMC Health Serv Res. 2016;16:367. 27507560 10.1186/s12913-016-1624-y PMC4979149

[27] Keskimäki I Tynkkynen L-K Reissell E Finland: health system review. Health Syst Transit. 2019;21:1–166. 31596240

[28] Parhiala K Avainlukuja perusterveydenhuollon järjestämisestä Suomessa 2013-2019 [in Finnish]. Helsinki: Finnish Institute of Health and Welfare; 2019:1–4.

[29] NHS Institute for Innovation and Improvement. Indicator construction: managing variation in emergency admissions. 2016. Available at: https://digital.nhs.uk/binaries/content/assets/website-assets/data-and-information/data-tools-and-services/data-services/innovative-uses-of-data/acsc-appendix-a.pdf. Accessed August 11, 2017.

[30] Blustein J Preventable hospitalizations and socioeconomic status. Health Affairs. 1998;17:177–189. 9558796 10.1377/hlthaff.17.2.177

[31] Willems S De Maesschalck S Deveugele M Socio-economic status of the patient and doctor-patient communication: does it make a difference? Patient Educ Counsel. 2005;56:139–146. 10.1016/j.pec.2004.02.011 15653242

[32] Buurman BM Frenkel WJ Abu-Hanna A Acute and chronic diseases as part of multimorbidity in acutely hospitalized older patients. Eur J Intern Med. 2016;27:68–75. 26477016 10.1016/j.ejim.2015.09.021

[33] Official Statistics of Finland: disability benefits [e-publication]. 2018. Available at: https://www.kela.fi/web/en/496. Accessed January 23, 2019.

[34] Delamater PL Messina JP Grady SC Do more hospital beds lead to higher hospitalization rates? A spatial examination of Roemer’s law. PLoS One. 2013;8:e54900. 23418432 10.1371/journal.pone.0054900 PMC3572098

[35] Mercer SW Zhou Y Humphris GM Multimorbidity and socioeconomic deprivation in primary care consultations. Ann Family Med. 2018;16:127–131. 10.1370/afm.2202 PMC5847350 29531103

[36] Pace M Lanzieri G Glickman M Revision of the European Standard Population: Report of Eurostat’s Task Force. Luxembourg: Publications Office of the European Union; 2013.

[37] Merlo J Yang M Chaix B A brief conceptual tutorial on multilevel analysis in social epidemiology: investigating contextual phenomena in different groups of people. J Epidemiol Community Health. 2005;59:729–736. 16100308 10.1136/jech.2004.023929 PMC1733145

[38] Austin PC Stryhn H Leckie G Measures of clustering and heterogeneity in multilevel Poisson regression analyses of rates/count data. Stat Med. 2018;37:572–589. 29114926 10.1002/sim.7532 PMC5813204

[39] R Core Team. R: A Language and Environment for Statistical Computing. Vienna, Austria: R Foundation for Statistical Computing; 2019.

[40] Bates D Mächler M Bolker B Fitting linear mixed-effects models using lme4. J Stat Softw. 2015;67:1–48.

[41] Paul MC Dik J-WH Hoekstra T Admissions for ambulatory care sensitive conditions: a national observational study in the general and COPD population. Eur J Public Health. 2019;29:213–219. 30212895 10.1093/eurpub/cky182 PMC6426039

[42] Falster MO Leyland AH Jorm LR Do hospitals influence geographic variation in admission for preventable hospitalisation? A data linkage study in New South Wales, Australia. BMJ Open. 2019;9:e027639. 10.1136/bmjopen-2018-027639 PMC6398792 30798320

[43] Berlin C Busato A Rosemann T Avoidable hospitalizations in Switzerland: a small area analysis on regional variation, density of physicians, hospital supply and rurality. BMC Health Serv Res. 2014;14:289. 24992827 10.1186/1472-6963-14-289 PMC4091658

[44] Kim AM Park JH Yoon TH Hospitalizations for ambulatory care sensitive conditions as an indicator of access to primary care and excess of bed supply. BMC Health Serv Res. 2019;19:259. 31029134 10.1186/s12913-019-4098-x PMC6487016

[45] Franks P Mooney C Sorbero M Physician referral rates: style without much substance? Med Care. 2000;38:836–846. 10929995 10.1097/00005650-200008000-00007

[46] Sund R Quality of the Finnish Hospital Discharge Register: a systematic review. Scand J Public Health. 2012;40:505–515. 22899561 10.1177/1403494812456637

[47] Ketola E Pitkänen V Huvinen S The national primary health care case-mix described [in Finnish]. Finnish Med J. 2019;74:2027–2034.

