# Supplementary material for: Individual and Area-level Factors Contributing to the Geographic Variation in Ambulatory Care Sensitive Conditions in Finland: A Register-based Study
Source: Med Care. 2020 Nov 16;59(2):123–30. doi: 10.1097/MLR.0000000000001454 (PMC7899221; doi:10.1097/MLR.0000000000001454)
Supplement: SUPPLEMENTARY MATERIAL [file mlr-59-123-s001.docx]

Supplemental Digital Content 1.

The Applied List of Ambulatory Care Sensitive Conditions (ACSCs) with ICD-10 Diagnosis Codes and Definition Notes.

| **ACSC conditions** | **ICD-10 diagnosis code** | **Definition notes** |
| --- | --- | --- |
| **Acute** |  |  |
| Cellulitis | L03, L04, L08.0, L08.8, L88, L98.0 | Primary diagnose only, exclude cases with other surgical procedures than skin procedures (A) |
| Convulsions and Epilepsy | G40, G41, O15, R56 | Primary diagnose only |
| Dehydration and Gastroenteritis | E86, K52.2, K52.8, K52.9 | Primary diagnose only |
| Dental Conditions | A69.0, K02-K06, K08, K09.8, K09.9, K12, K13 | Primary diagnose only |
| Gangrene | R02 | In any diagnosis field |
| Kidney and Urinary Tract Infections | N10, N11, N12, N13.6 | Primary diagnose only |
| Pelvic Inflammatory Disease | N70, N73, N74 | Primary diagnose only |
| Perforated or Bleeding Ulcer | K25.0-K25.2, K25.4-K25.6, K26.0-K26.2, K26.4-K26.6, K27.0-K27.2, K27.4-K27.6, K28.0-K28.2, K28.4-K28.6 | Primary diagnose only |
| Severe ENT infection | H66, H67, J02, J03, J06, J31.2 | Primary diagnose only |
| **Chronic** |  |  |
| Acute Bronchitis | J20 | Only when primary diagnose, and J41-J44 or J47 as a secondary diagnose |
| Angina | I20, I24.0, I24.8, I24.9 | Primary diagnose only, exclude cases with surgical procedures (B) |
| Asthma | J45, J46 | Primary diagnose only |
| Chronic Obstructive Pulmonary Disease (COPD) | J41, J42, J43, J44, J47 | Primary diagnose only |
| Congestive Heart Failure | I11.0, I50, J81 | Primary diagnose only, exclude cases with cardiac procedures (C) |
| Diabetes Complications | E10.0-10.8, E11.0-E11.8, E12.0-E12.8, E13.0-E13.8, E14.0-E14.8 | Primary diagnose only |
| Hypertension | I10, I11.9 | Primary diagnose only, exclude cases with cardiac procedures (C) |
| Iron Deficiency Anemia | D50.1-D50.9 | Primary diagnose only |
| Nutritional Deficiencies | E40, E41, E42, E43, E55.0, E64.3 | Primary diagnose only |
| **Vaccine-preventable** |  |  |
| Bacterial Pneumonia & Influenza | J09, J10, J11, J13, J14, J15.3, J15.4, J15.7, J15.9, J16.8, J18.1, J18.8, J18.9 | In any diagnosis field, do not accept if D57 is as a secondary diagnose |
| Immunization-Related and  Preventable Conditions | A35, A36, A37, A80, B05, B06, B16.1, B16.9, B18.0, B18.1, B26, G00.0, M01.4 | In any diagnosis field |

1. Cellulitis, no procedure field contains any of the following (NCSP, Nordic Classification of Surgical Procedures):

AA4WT, AA400, AA5LT, AAA-AAW, AB4AT, AB4BT, AB4CT, AB4DT, AB4FT, AB5, AB6AT, AB6BT, AB6CT, AB6DT, ABA-ABW, ACA-ACW, AD-AW, AXX90, BA3AT, BA4KT, BAA, BB2AT, BB3AT, BB4WT, BBA, BC1LT, BC2-BC4, BCA, BD-BW, CAA-CAW, CB, CCA-CCW, CD-CJ, CKA-CKW, CW, DAA-DAW, DB, DCA-DCW, DD, DEA-DEW, DFA-DFE, DFW99, DG, DHA-DLW, DMA-DPW, DQ-DW, EA, EBA-EBW, EC, EDA-EDW, EEA-EW, EF, EGA-EGW, EHA-EHW, EJ-EK, EL3AT, EL3RT, EL3YT, EL4 ,ELA-ELW, EMA-EMW, EP1AT, EP1LT, EP2AT, EP3AT, ENA-ENW, EW, FA, FBA-FBW, FCA-FCE, FD, FE1AT, FE2, FEA-FEW, FF-FL, FMA-FMW, FN1ST, FN1XT, FNA-FNW, FP-FX, GA2-GA4, GAA-GAW, GBA-GBW, GCA, GD1AT, GD1BT, GD1CT, GD1LT, GD2AT, GD2BT, GD2CT, GD3, GDA-GDW, GE1AT, GE1CT, GE1DT, GE2, GEA-GEW, GW, HA0, HA1AT, HA1DT, HA1MT, HA1ST, HA2-HA5, HAA-HAF, HW, JA1LT, JA2-JA3, JAA-JAW, JB, JCA-JCW, JDA-JDW, JE, JF3, JFA-JFW, JGA-JGW, JHA-JHW, JJ1AT, JJ2-JJ8, JJA-JJW, JK1-JK2, J3KAT, JK3BT, JK3CT, JK3FT, JK3LT, JK3NT, JK3RT, JK4-JK5, JKA-JKW, JL1-JL3, JLA-JLW, JM1AT, JM1LT, JM2, JMA-JMW, JN4LT, JW, JX1LT, JX1RT, JXA, KA2AT, KA3AT, KA3CT, KA3DT, KA3LT, KA4-KA6, KAA-KAW, KBA-KBW, KC1AT, KC2AT, KC3AT, KCA-KCW, KDA-KDW, KE1AT, KE1CT, KE2, KEA-KEW, KF1-KF7, KF8AT, KF8KT, KFA-KFW, KGA-KGW, KH1AT, KH1BT, KH1CT, KH1CT, KH1DT, KH1FT, KH1YT, KKA-KKW, KW, KX, LA1, LAA-LAW, LB1AT, LB1YT, LBA-LBW, LCA-LCW, LDA-LDW, LEA-LEW, LF-LW, LX1LT, MAA-MAW, MBA-MBW, MC-MW, NA0, NA6CT, NA7BT, NA7FT, NA7KT, NA7LT, NA9KT, NAA-NAW, NB1AT,NB1BT, NB1ZT, NB2, NBA-NBW, NCA-NCW, NDA-NDW, NEA-NEW, NFA-NFW, NGA-NGW, NHA-NHW, NJ3LT, NK1AT, NK1CT, NK1DT, NK1LT, NK2-NK3, NK4AT, NK4BT, NK5, NK6AT, NK6BT, NK6CT, NK6DT, NK6KT, NK7AT, NX, PA2ZT, PA3-PA5, PA6AT, PA8KT, PA9KT, PAA-PAW, PB1AT, PB1BT, PB1ST, PB1YT, PBA-PBW, PC2DT, PC2ET, PC5AT, PC5BT, PC5DT, PC5ET, PC5GT, PC5HT, PC5JT, PC5NT, PC5PT, PC5YT, PC6DT, PC6ET, PC6FT, PC7NT, PCA-PCW, PD1AT, PD1YT, PD2DT, PD3, PD4ST, PD5YT, PD6YT, PD7YT, PDA-PDW, PE, PG1AT, PG1BT, PG1ET-PG1LT, PG1MT-PG1VT, PG1YT-PG3YT, PG5RT-PG6NT, PGA-PGW, PH1AT, PH1FT, PH1UT, PH2ST, PH3YT, PH4AT, PH5GT, PH6GT, PH7FT, PH7UT, PH9AT, PH9ST, PH900, PHA-PHW, PJ2AT, PJ2CT, PJ2HT, PJ3-PJ4, PJ5AN, PJA-PJW, PW, PXA-PXX, QAA25, QAB00-QAB05, QAB99, QAC, QAD20, QAE-QAF, QAG10-QAG99, QAJ, QBA25, QBB00-QBB05, QBB99, QBC, QBD20, QBE, QBG10-QBG99, QBJ, QCA25, QCA30, QCB00-QCB05, QCB99, QCC, QCD20, QCE-QCG, QCJ, QDA25, QDB00-QDB05, QDB99, QDC, QDD20, QDE, QDG10-QDG99, QDJ, QXA25, QXB00-QXB05, QXB99, QXC, QXD20, QXE, QXG10-QXG99, QXJ, QX2ZT, QX3AT, QX3CT, QX3LT, QX3YT, QX4, S, TAA-TAD, TAW99, TA100, TBA-TJF, TJG10, TJJ, TJL-TJW, TK-TL, TMA-TPX, TQA-TQD, TQW00, TQW02, TQW30-40, TQW99, TQX00-TQX10, U, WW20, WW30-WW31, WW40, WW50, WX100-WX105, WX140-WX144, WX7-WX9, XCC00, XFE00, XFN96, XFX00, XFX10, XFX20, XFX97, XJW99, XPX00, XPX04, XPX08, XPX99, XW000, XW1-XW5, XX1AT, XX1BT, XX1CT, XX1DT, XX1XT, XX2AT-XX2DT, XX2XT, XX3AT-XX3DT, XX3XT, XX4-XX7, Y, ZC-ZP, ZS-ZX, ZZ

1. Angina, no procedure field contains any of the following:

AA4WT, AA400, AA5LT, AAA-AAW, AB4AT, AB4BT, AB4CT, AB4DT, AB4FT, AB5, AB6AT, AB6BT, AB6CT, AB6DT, ABA-ABW, ACA-ACW, AD-AW, AXX90, BA3AT, BA4KT, BAA, BB2AT, BB3AT, BB4WT, BBA, BC1LT, BC2-BC4, BCA, BD-BW, CAA-CAW, CB, CCA-CCW, CD-CJ, CKA-CKW, CW, DAA-DAW, DB, DCA-DCW, DD, DEA-DEW, DFA-DFE, DFW99, DG, DHA-DLW, DMA-DPW, DQ-DW, EA, EBA-EBW, EC, EDA-EDW, EEA-EW, EF, EGA-EGW, EHA-EHW, EJ-EK, EL3AT, EL3RT, EL3YT, EL4 ,ELA-ELW, EMA-EMW, EP1AT, EP1LT, EP2AT, EP3AT, ENA-ENW, EW, FA, FBA-FBW, FCA-FCE, FD, FE1AT, FE2, FEA-FEW, FF-FL, FMA-FMW, FN1ST, FN1XT, FNA-FNW, FP-FX, GA2-GA4, GAA-GAW, GBA-GBW, GCA, GD1AT, GD1BT, GD1CT, GD1LT, GD2AT, GD2BT, GD2CT, GD3, GDA-GDW, GE1AT, GE1CT, GE1DT, GE2, GEA-GEW, GW, HA0, HA1AT, HA1DT, HA1MT, HA1ST, HA2-HA5, HAA-HAF, HW, JA1LT, JA2-JA3, JAA-JAW, JB, JCA-JCW, JDA-JDW, JE, JF3, JFA-JFW, JGA-JGW, JHA-JHW, JJ1AT, JJ2-JJ8, JJA-JJW, JK1-JK2, J3KAT, JK3BT, JK3CT, JK3FT, JK3LT, JK3NT, JK3RT, JK4-JK5, JKA-JKW, JL1-JL3, JLA-JLW, JM1AT, JM1LT, JM2, JMA-JMW, JN4LT, JW, JX1LT, JX1RT, JXA, KA2AT, KA3AT, KA3CT, KA3DT, KA3LT, KA4-KA6, KAA-KAW, KBA-KBW, KC1AT, KC2AT, KC3AT, KCA-KCW, KDA-KDW, KE1AT, KE1CT, KE2, KEA-KEW, KF1-KF7, KF8AT, KF8KT, KFA-KFW, KGA-KGW, KH1AT, KH1BT, KH1CT, KH1CT, KH1DT, KH1FT, KH1YT, KKA-KKW, KW, KX, LA1, LAA-LAW, LB1AT, LB1YT, LBA-LBW, LCA-LCW, LDA-LDW, LEA-LEW, LF-LW, LX1LT, MAA-MAW, MBA-MBW, MC-MW, NA0, NA6CT, NA7BT, NA7FT, NA7KT, NA7LT, NA9KT, NAA-NAW, NB1AT,NB1BT, NB1ZT, NB2, NBA-NBW, NCA-NCW, NDA-NDW, NEA-NEW, NFA-NFW, NGA-NGW, NHA-NHW, NJ3LT, NK1AT, NK1CT, NK1DT, NK1LT, NK2-NK3, NK4AT, NK4BT, NK5, NK6AT, NK6BT, NK6CT, NK6DT, NK6KT, NK7AT, NX, PA2ZT, PA3-PA5, PA6AT, PA8KT, PA9KT, PAA-PAW, PB1AT, PB1BT, PB1ST, PB1YT, PBA-PBW, PC2DT, PC2ET, PC5AT, PC5BT, PC5DT, PC5ET, PC5GT, PC5HT, PC5JT, PC5NT, PC5PT, PC5YT, PC6DT, PC6ET, PC6FT, PC7NT, PCA-PCW, PD1AT, PD1YT, PD2DT, PD3, PD4ST, PD5YT, PD6YT, PD7YT, PDA-PDW, PE, PG1AT, PG1BT, PG1ET-PG1LT, PG1MT-PG1VT, PG1YT-PG3YT, PG5RT-PG6NT, PGA-PGW, PH1AT, PH1FT, PH1UT, PH2ST, PH3YT, PH4AT, PH5GT, PH6GT, PH7FT, PH7UT, PH9AT, PH9ST, PH900, PHA-PHW, PJ2AT, PJ2CT, PJ2HT, PJ3-PJ4, PJ5AN, PJA-PJW, PW, PXA-PXX, QAA-QAW, QBA-QBW, QCA-QCW, QDA-QDW, QW, QXA-QXW, QX2ZT, QX3AT, QX3CT, QX3LT, QX3YT, QX4, S, TAA-TAD, TAW99, TA100, TBA-TJF, TJG10, TJJ, TJL-TJW, TK-TL, TMA-TPX, TQ, U, WXQ, WW20, WW30-WW31, WW40, WW50, WX100-WX105, WX140-WX144, WX7-WX9, XCC00, XFE00, XFN96, XFX00, XFX10, XFX20, XFX97, XJW99, XPX00, XPX04, XPX08, XPX99, XW000, XW1-XW5, XX1AT, XX1BT, XX1CT, XX1DT, XX1XT, XX2AT-XX2DT, XX2XT, XX3AT-XX3DT, XX3XT, XX4-XX7, Y, ZC-ZP, ZS-ZX, ZZ

1. Congestive heart failure and hypertension, no procedure field contains any of the following:

FEA-FEW, FFA00, FFA10-FFA30, FFA96, FFB-FFW, FG-FH, FJA00, FJA96, FJB-FJW, FKA-FKW, FK1BT, FLA00, FLA96, FLB-FLW, FMA-FMW, FN1AT, FN1BT, FN1ST, FN1XT, FN1YT, FNA-FNW, FPA-FPF, FPH-FPW, FQ, FXA00-FXN00, TFN10, TFN99, TFP00, TFP40-TFP59

Incidence Rate Ratios of Individual and Area-level Factors with Total Ambulatory Care Sensitive Conditions (ACSCs) and ACSC subgroups in Finland in Three Time Periods; from the Multilevel Poisson Models Adjusted Simultaneously for All Individual and Area-level Factors (Model 6).

| **Total ACSCs** |  | 2011-12 | |  | 2013-14 | |  | 2015-17 | |
| --- | --- | --- | --- | --- | --- | --- | --- | --- | --- |
| Variable |  | IRR (CI 95%) | p-value |  | IRR (CI 95%) | p-value |  | IRR (CI 95%) | p-value |
| Gender |  |  |  |  |  |  |  |  |  |
| Male |  | 1.00 |  |  | 1.00 |  |  | 1.00 |  |
| Female |  | 0.71 (0.71-0.72) | **<0.001** |  | 0.73 (0.72-0.74) | **<0.001** |  | 0.74 (0.73-0.74) | **<0.001** |
| Age (years old) |  |  |  |  |  |  |  |  |  |
| 20-54 |  | 1.00 |  |  | 1.00 |  |  | 1.00 |  |
| 55-64 |  | 1.72 (1.69-1.76) | **<0.001** |  | 1.76 (1.72-1.79) | **<0.001** |  | 1.77 (1.74-1.80) | **<0.001** |
| 65-74 |  | 3.32 (3.26-3.39) | **<0.001** |  | 3.48 (3.41-3.55) | **<0.001** |  | 3.58 (3.52-3.63) | **<0.001** |
| 75-84 |  | 5.76 (5.66-5.87) | **<0.001** |  | 6.07 (5.96-6.19) | **<0.001** |  | 6.28 (6.18-6.37) | **<0.001** |
| 85+ |  | 10.72 (10.52-10.91) | **<0.001** |  | 11.31 (11.09-11.52) | **<0.001** |  | 12.06 (11.88-12.25) | **<0.001** |
| Income quintile |  |  |  |  |  |  |  |  |  |
| Lowest |  | 1.00 |  |  | 1.00 |  |  | 1.00 |  |
| 2 |  | 0.79 (0.78-0.80) | **<0.001** |  | 0.78 (0.77-0.79) | **<0.001** |  | 0.78 (0.77-0.78) | **<0.001** |
| 3 |  | 0.67 (0.67-0.68) | **<0.001** |  | 0.64 (0.63-0.65) | **<0.001** |  | 0.65 (0.64-0.65) | **<0.001** |
| 4 |  | 0.56 (0.56-0.57) | **<0.001** |  | 0.56 (0.55-0.57) | **<0.001** |  | 0.54 (0.53-0.55) | **<0.001** |
| Highest |  | 0.45 (0.44-0.46) | **<0.001** |  | 0.44 (0.43-0.45) | **<0.001** |  | 0.43 (0.43-0.44) | **<0.001** |
| No. comorbidities (+1 comorbidity) |  | 2.36 (2.35-2.37) | **<0.001** |  | 2.36 (2.35-2.37) | **<0.001** |  | 2.33 (2.32-2.33) | **<0.001** |
| Proportion of population aged 65+ receiving pensioner's care allowance (+1SD) |  | 1.12 (1.07-1.18) | **<0.001** |  | 1.07 (1.02-1.12) | **0.003** |  | 1.06 (1.02-1.11) | **0.008** |
| Proportion of ACSCs occurring in GP led wards of all ACSCs (+1SD) |  | 1.03 (1.00-1.06) | **0.044** |  | 1.02 (0.99-1.05) | 0.148 |  | 1.03 (1.00-1.07) | **0.024** |
| Rate of hospital bed utilization in specialist health care (+1SD) |  | 1.07 (1.03-1.11) | **<0.001** |  | 1.08 (1.04-1.12) | **<0.001** |  | 1.08 (1.04-1.12) | **<0.001** |
| Populations' average distance to emergency hospital (+1SD) |  | 0.98 (0.95-1.02) | 0.275 |  | 1.00 (0.97-1.03) | 0.995 |  | 1.00 (0.96-1.03) | 0.885 |
| Populations' average distance to health center (+1SD) |  | 1.01 (0.97-1.06) | 0.482 |  | 1.00 (0.96-1.04) | 0.906 |  | 1.00 (0.97-1.04) | 0.858 |
| Number of GPs per 1000 inhabitants (+1SD) |  | 1.00 (0.98-1.02) | 0.932 |  | 1.00 (0.98-1.02) | 0.942 |  | 1.00 (0.97-1.02) | 0.735 |
| Income median (+1SD) |  | 1.10 (1.05-1.14) | **<0.001** |  | 1.06 (1.02-1.10) | **0.007** |  | 1.07 (1.03-1.11) | **0.001** |

| **Acute ACSCs** |  | 2011-12 | |  | 2013-14 | |  | 2015-17 | |
| --- | --- | --- | --- | --- | --- | --- | --- | --- | --- |
| Variable |  | IRR (CI 95%) | p-value |  | IRR (CI 95%) | p-value |  | IRR (CI 95%) | p-value |
| Gender |  |  |  |  |  |  |  |  |  |
| Male |  | 1.00 |  |  | 1.00 |  |  | 1.00 |  |
| Female |  | 0.95 (0.93-0.97) | **<0.001** |  | 0.96 (0.94-0.97) | **<0.001** |  | 0.95 (0.93-0.96) | **<0.001** |
| Age (years old) |  |  |  |  |  |  |  |  |  |
| 20-54 |  | 1.00 |  |  | 1.00 |  |  | 1.00 |  |
| 55-64 |  | 1.09 (1.06-1.12) | **<0.001** |  | 1.08 (1.05-1.12) | **<0.001** |  | 1.11 (1.08-1.14) | **<0.001** |
| 65-74 |  | 1.50 (1.46-1.55) | **<0.001** |  | 1.56 (1.52-1.61) | **<0.001** |  | 1.66 (1.62-1.70) | **<0.001** |
| 75-84 |  | 2.23 (2.16-2.30) | **<0.001** |  | 2.34 (2.28-2.42) | **<0.001** |  | 2.47 (2.41-2.53) | **<0.001** |
| 85+ |  | 4.07 (3.95-4.19) | **<0.001** |  | 4.20 (4.08-4.33) | **<0.001** |  | 4.71 (4.60-4.83) | **<0.001** |
| Income quintile |  |  |  |  |  |  |  |  |  |
| Lowest |  | 1.00 |  |  | 1.00 |  |  | 1.00 |  |
| 2 |  | 0.76 (0.74-0.77) | **<0.001** |  | 0.72 (0.70-0.74) | **<0.001** |  | 0.74 (0.72-0.75) | **<0.001** |
| 3 |  | 0.65 (0.63-0.67) | **<0.001** |  | 0.62 (0.60-0.63) | **<0.001** |  | 0.63 (0.61-0.64) | **<0.001** |
| 4 |  | 0.54 (0.53-0.56) | **<0.001** |  | 0.54 (0.52-0.55) | **<0.001** |  | 0.53 (0.51-0.54) | **<0.001** |
| Highest |  | 0.45 (0.44-0.47) | **<0.001** |  | 0.45 (0.43-0.46) | **<0.001** |  | 0.44 (0.43-0.45) | **<0.001** |
| No. comorbidities (+1 comorbidity) |  | 1.90 (1.88-1.92) | **<0.001** |  | 1.93 (1.91-1.95) | **<0.001** |  | 1.89 (1.88-1.91) | **<0.001** |
| Proportion of population aged 65+ receiving pensioner's care allowance (+1SD) |  | 1.14 (1.07-1.22) | **<0.001** |  | 1.10 (1.03-1.17) | **0.003** |  | 1.12 (1.05-1.19) | **<0.001** |
| Proportion of ACSCs occurring in GP led wards of all ACSCs (+1SD) |  | 1.02 (0.98-1.06) | 0.330 |  | 1.03 (0.99-1.07) | 0.208 |  | 1.01 (0.97-1.05) | 0.622 |
| Rate of hospital bed utilization in specialist health care (+1SD) |  | 1.08 (1.02-1.14) | **0.006** |  | 1.06 (1.01-1.13) | **0.030** |  | 1.08 (1.03-1.14) | **0.002** |
| Populations' average distance to emergency hospital (+1SD) |  | 0.97 (0.92-1.02) | 0.242 |  | 0.96 (0.92-1.01) | 0.129 |  | 0.97 (0.92-1.02) | 0.254 |
| Populations' average distance to health center (+1SD) |  | 1.02 (0.97-1.08) | 0.423 |  | 1.02 (0.97-1.08) | 0.382 |  | 1.03 (0.98-1.08) | 0.285 |
| Number of GPs per 1000 inhabitants (+1SD) |  | 1.01 (0.98-1.04) | 0.635 |  | 1.02 (0.98-1.05) | 0.348 |  | 1.00 (0.96-1.03) | 0.850 |
| Income median (+1SD) |  | 1.16 (1.09-1.23) | **<0.001** |  | 1.08 (1.01-1.14) | **0.016** |  | 1.10 (1.05-1.16) | **<0.001** |

| **Chronic ACSCs** |  | 2011-12 | |  | 2013-14 | |  | 2015-17 | |
| --- | --- | --- | --- | --- | --- | --- | --- | --- | --- |
| Variable |  | IRR (CI 95%) | p-value |  | IRR (CI 95%) | p-value |  | IRR (CI 95%) | p-value |
| Gender |  |  |  |  |  |  |  |  |  |
| Male |  | 1.00 |  |  | 1.00 |  |  | 1.00 |  |
| Female |  | 0.71 (0.70-0.72) | **<0.001** |  | 0.75 (0.74-0.76) | **<0.001** |  | 0.75 (0.74-0.76) | **<0.001** |
| Age (years old) |  |  |  |  |  |  |  |  |  |
| 20-54 |  | 1.00 |  |  | 1.00 |  |  | 1.00 |  |
| 55-64 |  | 4.02 (3.84-4.21) | **<0.001** |  | 3.83 (3.65-4.01) | **<0.001** |  | 3.73 (3.59-3.88) | **<0.001** |
| 65-74 |  | 9.49 (9.08-9.92) | **<0.001** |  | 9.20 (8.78-9.63) | **<0.001** |  | 8.95 (8.62-9.29) | **<0.001** |
| 75-84 |  | 16.60 (15.88-17.34) | **<0.001** |  | 16.20 (15.48-16.96) | **<0.001** |  | 16.12 (15.54-16.73) | **<0.001** |
| 85+ |  | 28.56 (27.33-29.84) | **<0.001** |  | 28.63 (27.36-29.96) | **<0.001** |  | 28.69 (27.66-29.77) | **<0.001** |
| Income quintile |  |  |  |  |  |  |  |  |  |
| Lowest |  | 1.00 |  |  | 1.00 |  |  | 1.00 |  |
| 2 |  | 0.79 (0.78-0.80) | **<0.001** |  | 0.79 (0.78-0.80) | **<0.001** |  | 0.77 (0.76-0.78) | **<0.001** |
| 3 |  | 0.65 (0.64-0.67) | **<0.001** |  | 0.63 (0.62-0.64) | **<0.001** |  | 0.62 (0.61-0.63) | **<0.001** |
| 4 |  | 0.55 (0.54-0.56) | **<0.001** |  | 0.53 (0.52-0.54) | **<0.001** |  | 0.50 (0.49-0.51) | **<0.001** |
| Highest |  | 0.41 (0.39-0.42) | **<0.001** |  | 0.41 (0.39-0.42) | **<0.001** |  | 0.38 (0.37-0.39) | **<0.001** |
| No. comorbidities (+1 comorbidity) |  | 2.80 (2.79-2.82) | **<0.001** |  | 2.82 (2.80-2.83) | **<0.001** |  | 2.80 (2.79-2.81) | **<0.001** |
| Proportion of population aged 65+ receiving pensioner's care allowance (+1SD) |  | 1.06 (0.99-1.13) | 0.095 |  | 1.02 (0.95-1.08) | 0.625 |  | 1.00 (0.95-1.06) | 0.867 |
| Proportion of ACSCs occurring in GP led wards of all ACSCs (+1SD) |  | 1.03 (0.99-1.07) | 0.123 |  | 1.01 (0.97-1.06) | 0.506 |  | 1.04 (1.00-1.07) | 0.060 |
| Rate of hospital bed utilization in specialist health care (+1SD) |  | 1.11 (1.05-1.17) | **<0.001** |  | 1.13 (1.07-1.20) | **<0.001** |  | 1.12 (1.07-1.17) | **<0.001** |
| Populations' average distance to emergency hospital (+1SD) |  | 0.98 (0.94-1.03) | 0.492 |  | 1.02 (0.97-1.07) | 0.399 |  | 1.01 (0.97-1.06) | 0.568 |
| Populations' average distance to health center (+1SD) |  | 0.99 (0.94-1.05) | 0.835 |  | 0.98 (0.92-1.03) | 0.375 |  | 0.99 (0.94-1.03) | 0.564 |
| Number of GPs per 1000 inhabitants (+1SD) |  | 1.00 (0.97-1.03) | 0.779 |  | 0.99 (0.96-1.02) | 0.584 |  | 0.99 (0.96-1.02) | 0.515 |
| Income median (+1SD) |  | 1.05 (0.99-1.11) | 0.098 |  | 1.05 (0.99-1.11) | 0.134 |  | 1.05 (1.01-1.10) | **0.029** |

| **Vaccine-preventable ACSCs** |  | 2011-12 | |  | 2013-14 | |  | 2015-17 | |
| --- | --- | --- | --- | --- | --- | --- | --- | --- | --- |
| Variable |  | IRR (CI 95%) | p-value |  | IRR (CI 95%) | p-value |  | IRR (CI 95%) | p-value |
| Gender |  |  |  |  |  |  |  |  |  |
| Male |  | 1.00 |  |  | 1.00 |  |  | 1.00 |  |
| Female |  | 0.57 (0.56-0.58) | **<0.001** |  | 0.56 (0.56-0.57) | **<0.001** |  | 0.60 (0.59-0.61) | **<0.001** |
| Age (years old) |  |  |  |  |  |  |  |  |  |
| 20-54 |  | 1.00 |  |  | 1.00 |  |  | 1.00 |  |
| 55-64 |  | 1.96 (1.89-2.03) | **<0.001** |  | 2.34 (2.25-2.43) | **<0.001** |  | 2.16 (2.09-2.22) | **<0.001** |
| 65-74 |  | 4.12 (3.98-4.26) | **<0.001** |  | 5.19 (5.00-5.39) | **<0.001** |  | 4.88 (4.74-5.02) | **<0.001** |
| 75-84 |  | 7.81 (7.54-8.08) | **<0.001** |  | 9.96 (9.60-10.34) | **<0.001** |  | 9.20 (8.95-9.46) | **<0.001** |
| 85+ |  | 16.67 (16.12-17.24) | **<0.001** |  | 20.60 (19.85-21.37) | **<0.001** |  | 19.66 (19.13-20.21) | **<0.001** |
| Income quintile |  |  |  |  |  |  |  |  |  |
| Lowest |  | 1.00 |  |  | 1.00 |  |  | 1.00 |  |
| 2 |  | 0.82 (0.80-0.83) | **<0.001** |  | 0.79 (0.78-0.81) | **<0.001** |  | 0.80 (0.79-0.82) | **<0.001** |
| 3 |  | 0.72 (0.70-0.74) | **<0.001** |  | 0.67 (0.65-0.68) | **<0.001** |  | 0.69 (0.67-0.70) | **<0.001** |
| 4 |  | 0.59 (0.58-0.61) | **<0.001** |  | 0.59 (0.58-0.61) | **<0.001** |  | 0.59 (0.58-0.60) | **<0.001** |
| Highest |  | 0.49 (0.48-0.51) | **<0.001** |  | 0.46 (0.45-0.48) | **<0.001** |  | 0.48 (0.47-0.49) | **<0.001** |
| No. comorbidities (+1 comorbidity) |  | 2.01 (1.99-2.02) | **<0.001** |  | 2.03 (2.01-2.05) | **<0.001** |  | 2.01 (2.00-2.02) | **<0.001** |
| Proportion of population aged 65+ receiving pensioner's care allowance (+1SD) |  | 1.18 (1.12-1.24) | **<0.001** |  | 1.13 (1.07-1.19) | **<0.001** |  | 1.09 (1.04-1.15) | **0.001** |
| Proportion of ACSCs occurring in GP led wards of all ACSCs (+1SD) |  | 1.03 (1.00-1.06) | **0.049** |  | 1.03 (0.99-1.06) | 0.166 |  | 1.06 (1.02-1.10) | **0.002** |
| Rate of hospital bed utilization in specialist health care (+1SD) |  | 1.02 (0.98-1.07) | 0.333 |  | 1.04 (0.99-1.10) | 0.084 |  | 1.02 (0.98-1.07) | 0.325 |
| Populations' average distance to emergency hospital (+1SD) |  | 0.99 (0.95-1.03) | 0.630 |  | 1.01 (0.97-1.05) | 0.780 |  | 1.00 (0.95-1.04) | 0.926 |
| Populations' average distance to health center (+1SD) |  | 1.03 (0.99-1.08) | 0.162 |  | 1.00 (0.96-1.05) | 0.893 |  | 1.01 (0.96-1.06) | 0.792 |
| Number of GPs per 1000 inhabitants (+1SD) |  | 1.00 (0.97-1.02) | 0.950 |  | 1.00 (0.97-1.02) | 0.741 |  | 1.00 (0.96-1.03) | 0.839 |
| Income median (+1SD) |  | 1.10 (1.05-1.15) | **<0.001** |  | 1.08 (1.02-1.13) | **0.004** |  | 1.06 (1.01-1.11) | **0.020** |

Variance in Model with Only a Single Area-level Factor in Total Ambulatory Care Sensitive Conditions (ACSCs) and ACSC Subgroups between Health Center Areas (HC) and Hospital Districts (HD). Each Area-Level Factor Added First into Model 1 (Adjusted with Individual Age- and Sex) and Secondly into Model 2 (Adjusted with Individual Age, Sex, Socioeconomic Position and Health Status). PCV = Proportional Change in Area-Level Variance (PCV), Calculated as Percentual Decrease in Variance between Each Model and Model 1. MRR= Median Rate Ratio.

|  | **2011-12** | | | | | | | |  | | **2013-14** | | | | | | | |  | | **2015-17** | | | | | | | |
| --- | --- | --- | --- | --- | --- | --- | --- | --- | --- | --- | --- | --- | --- | --- | --- | --- | --- | --- | --- | --- | --- | --- | --- | --- | --- | --- | --- | --- |
|  | **HC** | | |  | **HD** | | |  | | **HC** | | | |  | **HD** | | |  | | **HC** | | | |  | **HD** | | |  |
|  | **σ^2^** | **PCV (%)** | **MRR** |  | **σ^2^** | **PCV (%)** | **MRR** |  | | **σ^2^** | | **PCV (%)** | **MRR** |  | **σ^2^** | **PCV (%)** | **MRR** |  | | **σ^2^** | | **PCV (%)** | **MRR** |  | **σ^2^** | **PCV (%)** | **MRR** |  |
| **Total ACSCs: area-level factors added separately into Model 1 (adjusted with individual age and sex = Null model)** | | | | | | | | | | | | | | | | | | | | | | | | | | | |  |
| Model 1 | 0.023 | - | 1.16 |  | 0.023 | - | 1.24 |  | | 0.020 | | - | 1.14 |  | 0.034 | - | 1.19 |  | | 0.019 | | - | 1.14 |  | 0.037 | - | 1.20 |  |
| Pensioner’s Care allowance | 0.016 | 31.6 | 1.13 |  | 0.021 | 57.4 | 1.15 |  | | 0.013 | | 31.4 | 1.12 |  | 0.015 | 54.5 | 1.13 |  | | 0.015 | | 20.6 | 1.13 |  | 0.017 | 52.1 | 1.13 |  |
| ACSCs in GP led wards | 0.021 | 8.9 | 1.15 |  | 0.037 | 25.0 | 1.20 |  | | 0.017 | | 14.1 | 1.13 |  | 0.024 | 29.6 | 1.16 |  | | 0.018 | | 9.5 | 1.13 |  | 0.025 | 31.6 | 1.16 |  |
| Hospital bed utilization rate | 0.016 | 29.7 | 1.13 |  | 0.030 | 38.7 | 1.18 |  | | 0.012 | | 38.0 | 1.11 |  | 0.015 | 57.1 | 1.12 |  | | 0.014 | | 26.7 | 1.12 |  | 0.018 | 49.4 | 1.14 |  |
| Distance to emergency | 0.023 | 1.4 | 1.15 |  | 0.045 | 10.2 | 1.22 |  | | 0.019 | | 3.9 | 1.14 |  | 0.027 | 19.1 | 1.17 |  | | 0.018 | | 5.1 | 1.14 |  | 0.030 | 18.0 | 1.18 |  |
| Distance to health center | 0.023 | 1.8 | 1.15 |  | 0.043 | 13.5 | 1.22 |  | | 0.019 | | 3.3 | 1.14 |  | 0.026 | 23.4 | 1.17 |  | | 0.019 | | 4.2 | 1.14 |  | 0.030 | 17.1 | 1.18 |  |
| Number of GPs | 0.023 | 2.1 | 1.15 |  | 0.045 | 9.0 | 1.23 |  | | 0.019 | | 3.2 | 1.14 |  | 0.027 | 19.0 | 1.17 |  | | 0.019 | | 2.2 | 1.14 |  | 0.031 | 15.5 | 1.18 |  |
| Income median | 0.020 | 12.1 | 1.15 |  | 0.041 | 18.0 | 1.21 |  | | 0.015 | | 22.0 | 1.12 |  | 0.025 | 25.1 | 1.16 |  | | 0.017 | | 14.8 | 1.13 |  | 0.029 | 20.0 | 1.18 |  |
| **Total ACSCs: area-level factors added separately into Model 2 (adjusted with individual age, sex, socioeconomic position and comorbidities)** | | | | | | | | | | | | | | | | | | | | | | | | | | | |  |
| Model 1 | 0.023 | - | 1.16 |  | 0.023 | - | 1.24 |  | | 0.020 | | - | 1.14 |  | 0.034 | - | 1.19 |  | | 0.019 | | - | 1.14 |  | 0.037 | - | 1.20 |  |
| Model 2 | 0.016 | 29.9 | 1.13 |  | 0.032 | 35.7 | 1.19 |  | | 0.014 | | 27.9 | 1.12 |  | 0.026 | 24.6 | 1.16 |  | | 0.016 | | 18.7 | 1.13 |  | 0.025 | 31.0 | 1.16 |  |
| Pensioner’s care allowance | 0.014 | 37.3 | 1.12 |  | 0.018 | 63.3 | 1.14 |  | | 0.013 | | 35.2 | 1.11 |  | 0.018 | 46.5 | 1.14 |  | | 0.015 | | 21.7 | 1.12 |  | 0.019 | 48.3 | 1.14 |  |
| ACSCs in GP led wards | 0.014 | 37.5 | 1.12 |  | 0.024 | 51.9 | 1.16 |  | | 0.012 | | 37.0 | 1.11 |  | 0.020 | 40.5 | 1.15 |  | | 0.014 | | 27.8 | 1.12 |  | 0.017 | 54.2 | 1.13 |  |
| Hospital bed utilization rate | 0.014 | 39.7 | 1.12 |  | 0.023 | 54.0 | 1.16 |  | | 0.012 | | 40.7 | 1.11 |  | 0.016 | 52.9 | 1.13 |  | | 0.014 | | 30.4 | 1.12 |  | 0.017 | 54.1 | 1.13 |  |
| Distance to emergency | 0.016 | 29.9 | 1.13 |  | 0.030 | 40.1 | 1.18 |  | | 0.014 | | 29.3 | 1.12 |  | 0.022 | 35.6 | 1.15 |  | | 0.015 | | 21.0 | 1.13 |  | 0.021 | 42.2 | 1.15 |  |
| Distance to health center | 0.016 | 29.6 | 1.13 |  | 0.030 | 40.3 | 1.18 |  | | 0.014 | | 27.9 | 1.12 |  | 0.022 | 34.1 | 1.15 |  | | 0.016 | | 19.4 | 1.13 |  | 0.022 | 39.2 | 1.15 |  |
| Number of GPs | 0.016 | 31.5 | 1.13 |  | 0.028 | 43.0 | 1.17 |  | | 0.014 | | 29.8 | 1.12 |  | 0.022 | 35.2 | 1.15 |  | | 0.015 | | 20.7 | 1.13 |  | 0.021 | 43.0 | 1.15 |  |
| Income median | 0.016 | 30.0 | 1.13 |  | 0.031 | 38.5 | 1.18 |  | | 0.014 | | 29.4 | 1.12 |  | 0.023 | 30.9 | 1.16 |  | | 0.016 | | 18.6 | 1.13 |  | 0.025 | 32.2 | 1.16 |  |

|  | **2011-12** | | | | | | | |  | | **2013-14** | | | | | | | |  | | **2015-17** | | | | | | | |
| --- | --- | --- | --- | --- | --- | --- | --- | --- | --- | --- | --- | --- | --- | --- | --- | --- | --- | --- | --- | --- | --- | --- | --- | --- | --- | --- | --- | --- |
|  | **HC** | | |  | **HD** | | |  | | **HC** | | | |  | **HD** | | |  | | **HC** | | | |  | **HD** | | |  |
|  | **σ^2^** | **PCV (%)** | **MRR** |  | **σ^2^** | **PCV (%)** | **MRR** |  | | **σ^2^** | | **PCV (%)** | **MRR** |  | **σ^2^** | **PCV (%)** | **MRR** |  | | **σ^2^** | | **PCV (%)** | **MRR** |  | **σ^2^** | **PCV (%)** | **MRR** |  |
| **Acute ACSCs: area-level factors added separately into Model 1 (adjusted with individual age and sex = Null model)** | | | | | | | | | | | | | | | | | | | | | | | | | | | |  |
| Model 1 | 0.027 | - | 1.17 |  | 0.038 | - | 1.21 |  | | 0.025 | | - | 1.16 |  | 0.035 | - | 1.20 |  | | 0.026 | | - | 1.17 |  | 0.055 | - | 1.25 |  |
| Pensioner’s Care allowance | 0.024 | 10.1 | 1.16 |  | 0.018 | 52.3 | 1.14 |  | | 0.021 | | 13.0 | 1.15 |  | 0.016 | 53.4 | 1.13 |  | | 0.022 | | 14.0 | 1.15 |  | 0.033 | 39.6 | 1.19 |  |
| ACSCs in GP led wards | 0.026 | 4.0 | 1.17 |  | 0.030 | 22.1 | 1.18 |  | | 0.023 | | 8.0 | 1.15 |  | 0.025 | 29.1 | 1.16 |  | | 0.025 | | 2.2 | 1.16 |  | 0.046 | 16.3 | 1.23 |  |
| Hospital bed utilization rate | 0.024 | 12.9 | 1.16 |  | 0.024 | 36.7 | 1.16 |  | | 0.021 | | 15.2 | 1.15 |  | 0.018 | 49.3 | 1.14 |  | | 0.023 | | 11.4 | 1.15 |  | 0.034 | 38.3 | 1.19 |  |
| Distance to emergency | 0.027 | 0.0 | 1.17 |  | 0.036 | 5.9 | 1.20 |  | | 0.024 | | 0.3 | 1.16 |  | 0.032 | 8.8 | 1.19 |  | | 0.025 | | 1.5 | 1.16 |  | 0.050 | 9.2 | 1.24 |  |
| Distance to health center | 0.027 | 0.4 | 1.17 |  | 0.035 | 9.2 | 1.19 |  | | 0.024 | | 1.2 | 1.16 |  | 0.030 | 14.5 | 1.18 |  | | 0.025 | | 2.5 | 1.16 |  | 0.049 | 10.4 | 1.24 |  |
| Number of GPs | 0.027 | 2.0 | 1.17 |  | 0.034 | 11.6 | 1.19 |  | | 0.024 | | 2.5 | 1.16 |  | 0.028 | 21.4 | 1.17 |  | | 0.026 | | 0.1 | 1.17 |  | 0.050 | 9.0 | 1.24 |  |
| Income median | 0.027 | 0.6 | 1.17 |  | 0.035 | 9.5 | 1.19 |  | | 0.023 | | 6.0 | 1.16 |  | 0.027 | 22.4 | 1.17 |  | | 0.025 | | 3.9 | 1.16 |  | 0.049 | 11.8 | 1.23 |  |
| **Acute ACSCs: area-level factors added separately into Model 2 (adjusted with individual age, sex, socioeconomic position and comorbidities)** | | | | | | | | | | | | | | | | | | | | | | | | | | | |  |
| Model 1 | 0.027 | - | 1.17 |  | 0.038 | - | 1.21 |  | | 0.025 | | - | 1.16 |  | 0.035 | - | 1.20 |  | | 0.026 | | - | 1.17 |  | 0.055 | - | 1.25 |  |
| Model 2 | 0.027 | 0.2 | 1.17 |  | 0.029 | 24.2 | 1.18 |  | | 0.022 | | 9.6 | 1.15 |  | 0.026 | 25.9 | 1.17 |  | | 0.024 | | 8.1 | 1.16 |  | 0.041 | 26.2 | 1.21 |  |
| Pensioner’s care allowance | 0.027 | 0.8 | 1.17 |  | 0.018 | 52.9 | 1.14 |  | | 0.022 | | 11.8 | 1.15 |  | 0.016 | 53.6 | 1.13 |  | | 0.023 | | 10.8 | 1.16 |  | 0.029 | 46.8 | 1.18 |  |
| ACSCs in GP led wards | 0.026 | 3.0 | 1.17 |  | 0.024 | 38.7 | 1.16 |  | | 0.021 | | 15.0 | 1.15 |  | 0.019 | 45.1 | 1.14 |  | | 0.023 | | 9.9 | 1.16 |  | 0.033 | 40.1 | 1.19 |  |
| Hospital bed utilization rate | 0.026 | 3.4 | 1.17 |  | 0.022 | 42.5 | 1.15 |  | | 0.021 | | 13.3 | 1.15 |  | 0.017 | 52.6 | 1.13 |  | | 0.023 | | 11.3 | 1.15 |  | 0.028 | 49.2 | 1.17 |  |
| Distance to emergency | 0.027 | 0.1 | 1.17 |  | 0.029 | 25.1 | 1.18 |  | | 0.022 | | 9.4 | 1.15 |  | 0.025 | 29.4 | 1.16 |  | | 0.024 | | 8.3 | 1.16 |  | 0.038 | 31.6 | 1.20 |  |
| Distance to health center | 0.027 | 0.0 | 1.17 |  | 0.028 | 25.9 | 1.17 |  | | 0.022 | | 9.3 | 1.15 |  | 0.024 | 32.3 | 1.16 |  | | 0.023 | | 8.6 | 1.16 |  | 0.038 | 31.9 | 1.20 |  |
| Number of GPs | 0.027 | 1.7 | 1.17 |  | 0.025 | 34.3 | 1.16 |  | | 0.022 | | 11.2 | 1.15 |  | 0.021 | 41.6 | 1.15 |  | | 0.024 | | 7.8 | 1.16 |  | 0.036 | 34.2 | 1.20 |  |
| Income median | 0.027 | 2.2 | 1.17 |  | 0.032 | 17.6 | 1.18 |  | | 0.022 | | 9.1 | 1.15 |  | 0.024 | 30.8 | 1.16 |  | | 0.024 | | 8.3 | 1.16 |  | 0.041 | 25.4 | 1.21 |  |

|  | **2011-12** | | | | | | | |  | | **2013-14** | | | | | | | |  | | **2015-17** | | | | | | | |
| --- | --- | --- | --- | --- | --- | --- | --- | --- | --- | --- | --- | --- | --- | --- | --- | --- | --- | --- | --- | --- | --- | --- | --- | --- | --- | --- | --- | --- |
|  | **HC** | | |  | **HD** | | |  | | **HC** | | | |  | **HD** | | |  | | **HC** | | | |  | **HD** | | |  |
|  | **σ^2^** | **PCV (%)** | **MRR** |  | **σ^2^** | **PCV (%)** | **MRR** |  | | **σ^2^** | | **PCV (%)** | **MRR** |  | **σ^2^** | **PCV (%)** | **MRR** |  | | **σ^2^** | | **PCV (%)** | **MRR** |  | **σ^2^** | **PCV (%)** | **MRR** |  |
| **Chronic ACSCs: area-level factors added separately into Model 1 (adjusted with individual age and sex = Null model)** | | | | | | | | | | | | | | | | | | | | | | | | | | | |  |
| Model 1 | 0.034 | - | 1.19 |  | 0.072 | - | 1.29 |  | | 0.029 | | - | 1.18 |  | 0.055 | - | 1.25 |  | | 0.027 | | - | 1.17 |  | 0.035 | - | 1.20 |  |
| Pensioner’s Care allowance | 0.027 | 20.2 | 1.17 |  | 0.035 | 51.5 | 1.,2 |  | | 0.024 | | 17.3 | 1.16 |  | 0.030 | 45.1 | 1.18 |  | | 0.024 | | 9.9 | 1.16 |  | 0.016 | 54.5 | 1.13 |  |
| ACSCs in GP led wards | 0.032 | 6.3 | 1.19 |  | 0.057 | 21.1 | 1.26 |  | | 0.027 | | 9.4 | 1.17 |  | 0.044 | 19.9 | 1.22 |  | | 0.025 | | 7.7 | 1.16 |  | 0.025 | 29.1 | 1.16 |  |
| Hospital bed utilization rate | 0.023 | 33.0 | 1.15 |  | 0.039 | 46.7 | 1.21 |  | | 0.017 | | 42.3 | 1.13 |  | 0.022 | 60.8 | 1.15 |  | | 0.017 | | 36.3 | 1.13 |  | 0.013 | 62.3 | 1.12 |  |
| Distance to emergency | 0.033 | 1.2 | 1.19 |  | 0.064 | 11.4 | 1.27 |  | | 0.028 | | 4.7 | 1.17 |  | 0.045 | 19.3 | 1.22 |  | | 0.025 | | 6.5 | 1.16 |  | 0.026 | 25.5 | 1.17 |  |
| Distance to health center | 0.034 | 0.9 | 1.19 |  | 0.061 | 15.4 | 1.27 |  | | 0.029 | | 2.9 | 1.17 |  | 0.042 | 24.1 | 1.22 |  | | 0.026 | | 4.1 | 1.17 |  | 0.025 | 29.3 | 1.16 |  |
| Number of GPs | 0.034 | 1.0 | 1.19 |  | 0.067 | 6.9 | 1.28 |  | | 0.029 | | 1.9 | 1.18 |  | 0.048 | 13.7 | 1.23 |  | | 0.026 | | 2.4 | 1.17 |  | 0.030 | 15.1 | 1.18 |  |
| Income median | 0.029 | 15.6 | 1.18 |  | 0.054 | 25.5 | 1.25 |  | | 0.023 | | 22.9 | 1.15 |  | 0.040 | 27.5 | 1.21 |  | | 0.022 | | 16.8 | 1.15 |  | 0.024 | 30.9 | 1.16 |  |
| **Chronic ACSCs: area-level factors added separately into Model 2 (adjusted with individual age, sex, socioeconomic position and comorbidities)** | | | | | | | | | | | | | | | | | | | | | | | | | | | |  |
| Model 1 | 0.034 | - | 1.19 |  | 0.072 | - | 1.29 |  | | 0.029 | | - | 1.18 |  | 0.055 | - | 1.25 |  | | 0.027 | | - | 1.17 |  | 0.035 | - | 1.20 |  |
| Model 2 | 0.024 | 28.9 | 1.16 |  | 0.049 | 32.6 | 1.23 |  | | 0.025 | | 13.4 | 1.16 |  | 0.044 | 21.5 | 1.22 |  | | 0.023 | | 13.9 | 1.16 |  | 0.026 | 27.6 | 1.16 |  |
| Pensioner’s care allowance | 0.023 | 31.8 | 1.16 |  | 0.033 | 55.0 | 1.19 |  | | 0.025 | | 14.9 | 1.16 |  | 0.035 | 36.7 | 1.20 |  | | 0.023 | | 13.3 | 1.16 |  | 0.022 | 38.5 | 1.15 |  |
| ACSCs in GP led wards | 0.022 | 34.9 | 1.15 |  | 0.039 | 46.0 | 1.21 |  | | 0.024 | | 19.4 | 1.16 |  | 0.038 | 31.2 | 1.20 |  | | 0.021 | | 21.8 | 1.15 |  | 0.019 | 46.8 | 1.14 |  |
| Hospital bed utilization rate | 0.020 | 41.2 | 1.14 |  | 0.031 | 57.1 | 1.18 |  | | 0.021 | | 29.2 | 1.15 |  | 0.026 | 53.9 | 1.16 |  | | 0.018 | | 31.9 | 1.14 |  | 0.017 | 52.3 | 1.13 |  |
| Distance to emergency | 0.024 | 28.9 | 1.16 |  | 0.045 | 38.3 | 1.22 |  | | 0.025 | | 15.6 | 1.16 |  | 0.036 | 34.3 | 1.20 |  | | 0.022 | | 17.9 | 1.15 |  | 0.020 | 44.3 | 1.14 |  |
| Distance to health center | 0.024 | 28.4 | 1.16 |  | 0.045 | 38.3 | 1.22 |  | | 0.026 | | 13.1 | 1.16 |  | 0.038 | 32.1 | 1.20 |  | | 0.023 | | 14.3 | 1.16 |  | 0.020 | 42.6 | 1.15 |  |
| Number of GPs | 0.024 | 29.7 | 1.16 |  | 0.044 | 38.5 | 1.22 |  | | 0.025 | | 14.5 | 1.16 |  | 0.039 | 29.0 | 1.21 |  | | 0.023 | | 16.5 | 1.15 |  | 0.022 | 38.4 | 1.15 |  |
| Income median | 0.024 | 30.4 | 1.16 |  | 0.043 | 41.0 | 1.22 |  | | 0.025 | | 15.8 | 1.16 |  | 0.039 | 29.9 | 1.21 |  | | 0.023 | | 13.7 | 1.16 |  | 0.024 | 30.7 | 1.16 |  |

|  | **2011-12** | | | | | | | |  | | **2013-14** | | | | | | | |  | | **2015-17** | | | | | | | |
| --- | --- | --- | --- | --- | --- | --- | --- | --- | --- | --- | --- | --- | --- | --- | --- | --- | --- | --- | --- | --- | --- | --- | --- | --- | --- | --- | --- | --- |
|  | **HC** | | |  | **HD** | | |  | | **HC** | | | |  | **HD** | | |  | | **HC** | | | |  | **HD** | | |  |
|  | **σ^2^** | **PCV (%)** | **MRR** |  | **σ^2^** | **PCV (%)** | **MRR** |  | | **σ^2^** | | **PCV (%)** | **MRR** |  | **σ^2^** | **PCV (%)** | **MRR** |  | | **σ^2^** | | **PCV (%)** | **MRR** |  | **σ^2^** | **PCV (%)** | **MRR** |  |
| **Vaccine-preventable ACSCs: area-level factors added separately into Model 1 (adjusted with individual age and sex = Null model)** | | | | | | | | | | | | | | | | | | | | | | | | | | | |  |
| Model 1 | 0.024 | - | 1.16 |  | 0.033 | - | 1.19 |  | | 0.026 | | - | 1.17 |  | 0.021 | - | 1.15 |  | | 0.024 | | - | 1.16 |  | 0.037 | - | 1.20 |  |
| Pensioner’s Care allowance | 0.015 | 39.2 | 1.12 |  | 0.019 | 42.1 | 1.14 |  | | 0.017 | | 33.9 | 1.13 |  | 0.015 | 27.3 | 1.12 |  | | 0.019 | | 21.9 | 1.14 |  | 0.022 | 40.2 | 1.15 |  |
| ACSCs in GP led wards | 0.022 | 9.8 | 1.15 |  | 0.023 | 29.8 | 1.16 |  | | 0.023 | | 11.6 | 1.16 |  | 0.014 | 31.8 | 1.12 |  | | 0.022 | | 11.2 | 1.15 |  | 0.023 | 38.3 | 1.16 |  |
| Hospital bed utilization rate | 0.019 | 21.4 | 1.14 |  | 0.027 | 17.0 | 1.17 |  | | 0.020 | | 23.0 | 1.15 |  | 0.016 | 25.4 | 1.13 |  | | 0.022 | | 11.3 | 1.15 |  | 0.026 | 30.5 | 1.17 |  |
| Distance to emergency | 0.023 | 4.7 | 1.16 |  | 0.029 | 12.8 | 1.18 |  | | 0.025 | | 4.2 | 1.16 |  | 0.017 | 20.4 | 1.13 |  | | 0.024 | | 3.1 | 1.16 |  | 0.032 | 13.8 | 1.19 |  |
| Distance to health center | 0.023 | 6.3 | 1.15 |  | 0.028 | 14.5 | 1.17 |  | | 0.025 | | 4.1 | 1.16 |  | 0.017 | 18.6 | 1.13 |  | | 0.024 | | 3.6 | 1.16 |  | 0.034 | 9.4 | 1.19 |  |
| Number of GPs | 0.024 | 2.2 | 1.16 |  | 0.031 | 7.5 | 1.18 |  | | 0.026 | | 2.3 | 1.17 |  | 0.018 | 14.5 | 1.14 |  | | 0.024 | | 1.5 | 1.16 |  | 0.032 | 14.0 | 1.19 |  |
| Income median | 0.021 | 12.8 | 1.15 |  | 0.031 | 7.4 | 1.18 |  | | 0.023 | | 13.4 | 1.16 |  | 0.019 | 7.9 | 1.14 |  | | 0.022 | | 9.6 | 1.15 |  | 0.032 | 12.4 | 1.19 |  |
| **Vaccine-preventable ACSCs: area-level factors added separately into Model 2 (adjusted with individual age, sex, socioeconomic position and comorbidities)** | | | | | | | | | | | | | | | | | | | | | | | | | | | |  |
| Model 1 | 0.024 | - | 1.16 |  | 0.033 | - | 1.19 |  | | 0.026 | | - | 1.17 |  | 0.021 | - | 1.15 |  | | 0.024 | | - | 1.16 |  | 0.037 | - | 1.20 |  |
| Model 2 | 0.018 | 25.9 | 1.14 |  | 0.026 | 20.1 | 1.17 |  | | 0.020 | | 26.1 | 1.14 |  | 0.021 | 1.4 | 1.15 |  | | 0.021 | | 13.1 | 1.15 |  | 0.032 | 14.8 | 1.18 |  |
| Pensioner’s care allowance | 0.014 | 43.0 | 1.12 |  | 0.019 | 42.2 | 1.14 |  | | 0.016 | | 40.1 | 1.13 |  | 0.019 | 9.6 | 1.14 |  | | 0.020 | | 19.9 | 1.14 |  | 0.026 | 31.0 | 1.16 |  |
| ACSCs in GP led wards | 0.016 | 34.0 | 1.13 |  | 0.020 | 38.5 | 1.15 |  | | 0.017 | | 34.3 | 1.13 |  | 0.017 | 18.7 | 1.13 |  | | 0.019 | | 23.8 | 1.14 |  | 0.021 | 44.1 | 1.15 |  |
| Hospital bed utilization rate | 0.016 | 33.2 | 1.13 |  | 0.025 | 25.8 | 1.16 |  | | 0.017 | | 34.2 | 1.13 |  | 0.019 | 10.8 | 1.14 |  | | 0.020 | | 17.0 | 1.15 |  | 0.027 | 27.6 | 1.17 |  |
| Distance to emergency | 0.018 | 27.8 | 1.13 |  | 0.024 | 27.4 | 1.16 |  | | 0.019 | | 28.2 | 1.14 |  | 0.018 | 13.1 | 1.14 |  | | 0.021 | | 14.4 | 1.15 |  | 0.029 | 23.0 | 1.17 |  |
| Distance to health center | 0.018 | 27.5 | 1.13 |  | 0.024 | 27.9 | 1.16 |  | | 0.019 | | 26.8 | 1.14 |  | 0.019 | 9.8 | 1.14 |  | | 0.021 | | 13.9 | 1.15 |  | 0.030 | 18.9 | 1.18 |  |
| Number of GPs | 0.018 | 27.4 | 1.13 |  | 0.025 | 25.7 | 1.16 |  | | 0.019 | | 27.5 | 1.14 |  | 0.019 | 8.2 | 1.14 |  | | 0.021 | | 14.3 | 1.15 |  | 0.028 | 25.3 | 1.17 |  |
| Income median | 0.018 | 27.3 | 1.14 |  | 0.026 | 21.1 | 1.17 |  | | 0.019 | | 27.3 | 1.14 |  | 0.020 | 2.6 | 1.15 |  | | 0.021 | | 13.2 | 1.15 |  | 0.031 | 15.9 | 1.18 |  |
